# Tandem microfluidic chip isolation of prostate and breast cancer cells from simulated liquid biopsies using CD71 as an affinity ligand†

**DOI:** 10.1039/d0ra03626a

**Published:** 2020-09-02

**Authors:** Bhagya Wickramaratne, Dimitri Pappas

**Affiliations:** Department of Chemistry and Biochemistry, Texas Tech University Lubbock TX USA d.pappas@ttu.edu

## Abstract

The use of blood as a liquid biopsy provides a minimally invasive and less traumatic approach for initial cancer screens as well as patient monitoring. However, current clinical protocols require *a priori* knowledge of cancer type for liquid biopsy analyses. Previously, we proposed the use of the human transferrin 1 receptor protein (CD71) as a universal capture target for cancer cells analyses. In this study we have attempted to identify the lowest limit of detection for circulating tumor cells of prostate (PC-3) and breast cancers (MDA-MB-231) using CD71. We used a novel high-throughput herringbone chip design which could extract PC-3 cells at 34 ± 5% purity and MDA-MB-231 cells at 43 ± 35% purity when spiked to lysed blood at 0.1%. MDA-MB-231 cell spiked samples showed higher standard deviation, but the system captured 55 ± 16 cells, which is a sufficient number of cells for subsequent analyses. Further, this herringbone chip design has been shown to be compatible with an erythrocyte lysis chip we have described in previous studies. This circuit was capable of capturing 510 ± 120 cells with a purity of 82 ± 14% using <7 μL of a whole blood sample spiked with 10% MDA-MB-231 cells. Using an erythrocyte lysis circuit eliminates the need for human intervention for target cell enrichment, thereby reducing cell loss and sample contamination. We have shown that, when used with the high-throughput herringbone chip CD71 has the capacity to sensitively detect rare target cells for routine low-cost cancer screens.

## Abstract 2

The use of blood as a liquid biopsy provides a minimally invasive and less traumatic approach for initial cancer screens as well as patient monitoring.

## Graphical abstract

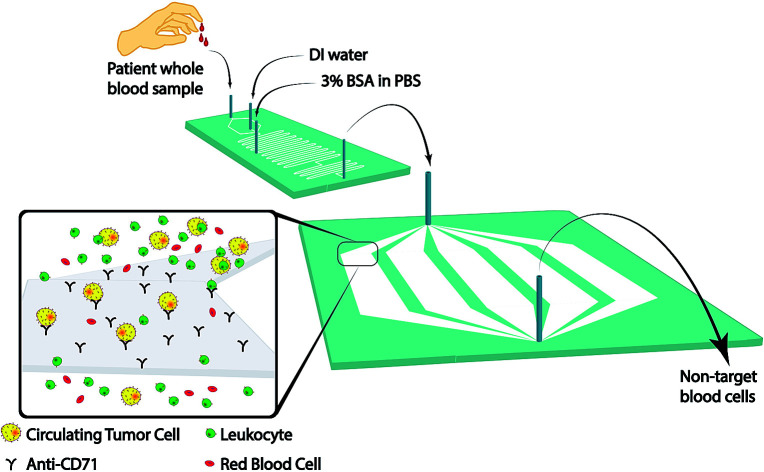

## 1 Introduction

Cancer is the second leading cause of death worldwide and the cost of cancer-related care was estimated to be over 100 billion dollars in the United States in 2017 alone.^1^ However, advances in treatment options and technology have attributed to a decline in cancer mortality.^2^ Typical cancer treatments include chemotherapy, hormone therapy, target therapy drugs, immunotherapy and surgery.^3^ These treatments are prescribed to patients based on the results of a tissue biopsy, which is the current gold standard for diagnosing cancer. Tissue biopsy is an invasive procedure requiring a sample of the suspect tissue to be surgically removed or aspirated, and observed under a microscope for abnormalities.^4^ While aspiration is less invasive,^5^ both these methods increase the risk of tumor metastasis.^4^ Due to risks involved with biopsies, prolonged patient treatments are mostly based on the initial biopsy report. However, since cancerous tissue may change their physiology over time, the patient may ultimately become unresponsive to treatment.^6^ Liquid biopsies are less invasive, therefore provide a more attractive alternative for the initial diagnosis as well as patient monitoring. Depending on the patient's health condition, blood provides a readily available liquid biopsy for monitoring effectiveness of treatment, and allows researchers to study changes in cancer cells.^6^

One of the main challenges of using liquid biopsies for cancer analyses is the low number of circulating tumor cells (CTCs).^6^ During the early stages of a cancer CTC concentrations can be as low as 1–1000 cells in 10 mL of blood.^6,7^ Therefore, CTCs must be enriched prior to analyses. Such enrichment techniques include methods based on cell size,^8^ deformability, electrical charge on cell surface,^9^ microfiltration, centrifugation, differential inertial focusing,^10–13^ microvortices,^14^ Dean flow,^15,16^ immunoaffinity methods, and density gradient separation.^17^ Some studies focus on detecting cell clusters, which demonstrate higher metastatic propensity, rather than single cells.^18^ A few researchers have attempted gene sequencing using tumor derived DNA in blood samples.^19^ Of these techniques immunoaffinity based cell capture *via* antibodies and aptamers is the most widely adopted.^18^

Immunoaffinity techniques are capable of achieving high capture purities and efficiencies.^18^ CellSearch® is such a platform and is the only technology approved by FDA to be used for the clinical analysis of liquid biopsies.^20,21^ This system depends on the expression of the epithelial cell adhesion marker (EpCAM) to detect CTCs.^20^ However, EpCAM is only expressed on tumor cells of epithelial origin, such as prostate and breast cancer cells. EpCAM is not present in cells of hematopoietic or mesenchymal origin, and is downregulated in CTCs of epithelial origin which have undergone the epithelial-to-mesenchymal transition.^21^ Therefore, although EpCAM is the extant gold standard, it cannot serve as a pan-cancer screening affinity ligand. In previous studies we have proposed the use of the human transferrin 1 receptor (CD71) as target antigen for cancer screening without *a priori* knowledge of cancer type due to its high expression in cancer cells and low expression in healthy blood cells. Cancer cells proliferate indefinitely therefore their expression of CD71 is maintained at a significantly higher level than healthy blood cells at any time of the cell cycle.^22^ These studies have shown that anti-CD71 can effectively be used to detect acute myeloid leukemia cells^23^ and acute lymphoblastic leukemia cells^24^ below the WHO threshold. In this study we tested the utility of CD71 as a capture target for prostate cancer (PC-3) and triple-negative breast cancer (MDA-MB-231). Currently used affinity ligands include anti-PSMA, prostatic acid phosphatase,^25^ and triple stain (combination of antibodies for P504S, p63, and CK903)^26^ for prostate tissue. Estrogen receptors (ER) and progesterone receptors (PR), and HER2 biomarkers are used for the detection and treatment of breast cancer.^24,27^ Triple negative breast cancers, which account for 10–20% of breast cancers, do not express ER, PR or HER2 receptors.^28–30^

Prostate cancer is the 5^th^ leading cause of death in men, worldwide. Initial screens for prostate cancer include digital rectal examination (DRE) and measurement of serum prostate specific antigen (PSA).^31^ Neither of these methods are highly specific or predictive, so they are used in combination with other symptoms and family history before a patient is suspected to have cancer.^26,32^ Globally, invasive breast cancer is the most common type of cancer affecting women, but it has a good prognosis if detected early. Only 1% of breast cancers account for those in men.^33^ Mammography screening and MRI screening are used to detect tumors at the early stages.^34^

We propose the use of anti-CD71 in combination with a high throughput 5-channel herringbone chip design which has a detection limit of two orders of magnitude lower than we have previously reported for standard herringbone devices,^23,24^ and an order of magnitude lower than nanoparticle coated herringbone chips.^35^ This chip can detect PC-3 and MDA-MB-231 cells at a 0.1% spike in lysed blood, and has several advantages over a single channel herringbone chip. Further, this chip is compatible with the on-chip lysis circuit we have described previously,^36^ enabling analysis of whole blood with minimal preprocessing.

## 2 Materials and methods

### 2.1 Experimental setup

**Fig. 1:**
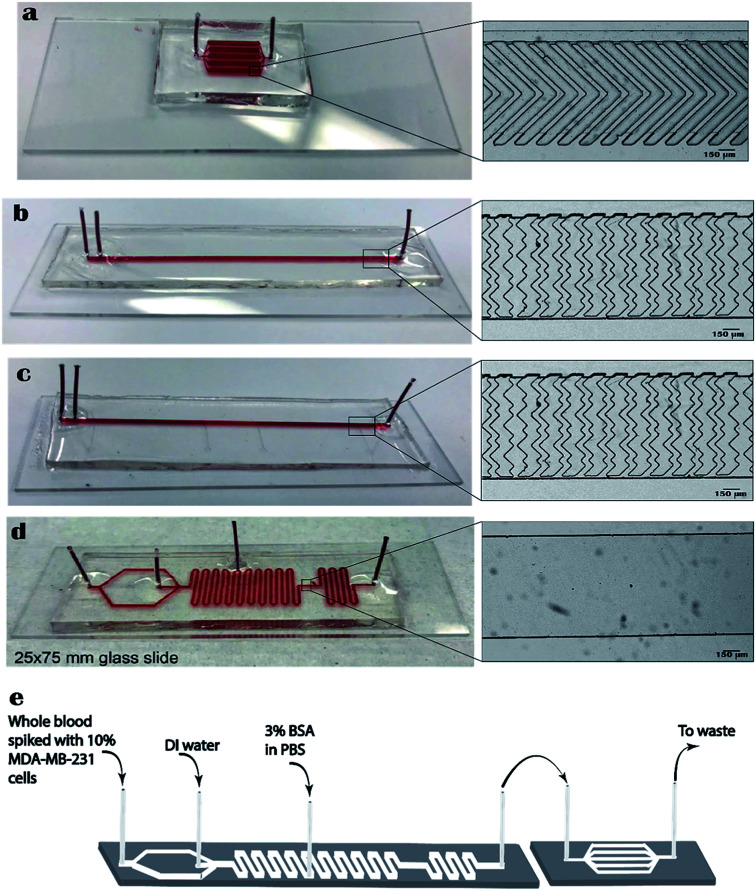
Images of PDMS chips bonded to glass slides used for analyses. Food coloring has been injected to visualize channels. The enlarged image on the right is microscope image of the channel. All glass slides are 25 × 75 × 1 mm in size. (a) 5-Channel herringbone chip used for MDA-MB-231 concentration study. All 5 channels have similar herringbone structures. (b) 5 cm single-channel herringbone chip used for PC-3 flow rate study. (c) 2 cm single-channel herringbone chip used for PC-3 concentration study. 2 cm refers to the length of channel having herringbone structures (refer Fig. 2b). (d) Lysis and buffering chip (L&B chip). (e) A schematic of the whole blood setup for the on-chip lysis study. The parallelly run lysed blood sample was directly injected to the 5-channel herringbone chip.

For all studies using lysed blood, the sample was directly pumped into the inlet of the herringbone chip using a syringe pump. In studies using whole blood, the sample was pumped into the lysis and buffering chip (L&B chip) which was directly connected to the 5-channel herringbone chip (Fig. 1a and e). More details on the L&B chip can be found in ref. 36. At the end of each run the cells were washed with 3% BSA in PBS (wash buffer) to remove unbound or weakly bound cells. All experiments were carried out in triplicate.

### 2.2 Cell culture and preparation

Human prostate adenocarcinoma cells (PC-3), and human breast adenocarcinoma (MDA-MB-231) cells were purchased from American Type Culture Collection (ATCC). Each of these cells were cultured in 25 mL of RPMI 1640 medium (Hyclone) and incubated at 37 °C at 5% CO_2_. Prior to use, each liter of RPMI 1640 medium was supplemented with 20 mL of penicillin-streptomycin stabilized solution (Sigma-Aldrich) with 10% fetal bovine serum (Hyclone). PC-3 and MDA-MB-231 cell types were subcultured 2–3 days before measurements to maintain CD71 expression. PC-3 cell suspension was extracted from the culture flask^37^ and centrifuged at 4500 rpm for 5 min. The supernatant was removed from the centrifuged sample, and the cell pellet was resuspended in 1000 μL of RPMI medium. The cells were stained by incubating with MitoTracker Green at 37 °C in 5% CO_2_ for 45 min. The cells were washed 5 times post-staining using phosphate buffered saline (PBS, VWR, pH 7.4) and the final cell pellet was resuspended in 3% bovine serum albumin (BSA) in PBS. The cell counts were determined using a hemocytometer. The same procedure was followed to prepare MDA-MB-231 cells for experiments.

### 2.3 Preparation of blood samples

Commercial whole blood (Multi-Check Control, Becton-Dickinson) was stained with 1 μL of propidium iodide (PI) for every 100 μL of blood. For experiments using lysed blood, whole blood was lysed by mixing with 900 μL of DI water for 30 s and restoring osmolarity with 110 μL of 80 g mL^−1^ NaCl solution, for every 100 μL of whole blood. The lysed blood sample was centrifuged at 4500 rpm for 5 min. The resulting pellet was washed three times with PBS and the final pellet was resuspended in 3% BSA in PBS. Stained blood was used as is for whole blood experiments. Studies comparing capture from on-chip lysis and pre-lysed blood were conducted using two aliquots from same initial sample. Blood samples were acquired from a commercial source (Becton-Dickinson) and are de-identified.

### 2.4 Preparation of spiked blood samples

PC-3, MDA-MB-231, and white blood cell concentrations were determined using a hemocytometer. The respective spike volume was calculated, and target cells were spiked to blood sample immediately before each run. The number of cancer cells spiked were calculated as a percentage of leukocytes. Cell concentrations are stated in Table 1.

**Table 1:** Purity and enrichment factors for various spiked concentrations of PC-3 and MDA-MB-231 cells (*n* = 3)

Experiment		Cancer cells		Purity of captured cancer cells		Enrichment	
Method no.	Spike%	Concentration in blood (cells per μL)	Standard deviation	%	Standard deviation	Enrichment factor	Standard deviation
3.2 – 2 cm single-channel chip PC-3 cells|0.04 mL h^−1^	20	336	133	94	7	5	0.4
	10	133	7	64	22	6	2
	1	17	1	56	22	56	22
	0.5	8	1	12	12	24	24
	0.1	3	0.02	6	4	46	32
3.2 – 2 cm single-channel chip PC-3 cells|0.12 mL h^−1^	0.5	8	0.1	25	6	49	13
	0.1	1	0.2	2	1	21	9
3.2 – 5-channel chip PC-3 cells|0.2 mL h^−1^	0.1	2	0	34	5	339	48
3.3 – 5-channel chip MDA-MB-231 cells|0.2 mL h^−1^	10	176	10	93	6	9	1
	1	17	1	72	23	72	23
	0.5	7	1	63	30	125	60
	0.1	2	0	43	35	432	352

### 2.5 Fabrication of microfluidic device

A 4-inch diameter silicon wafer (University Wafer) was spin coated with negative photoresist (SU-8 2015, Micro Chem) at 1000 rpm for 30 s to achieve a 40 μm thick coating of polymer. This wafer then underwent a prebake at 95 °C for 5 min followed by exposure to ultraviolet light under a high-resolution mask designed with Adobe Illustrator (20 000 dpi laser printer transparency by CAD Art Services). UV treatment of the wafer was followed by a postbake at 95 °C for 5 min. The wafer was then washed with SU-8 developer (Micro Chem) and 2-propanol (Fisher Chemical) to remove excess photoresist and baked for a further 10 min at 200 °C. Finally, the wafer was made hydrophobic by incubating in 1*H*,1*H*,2*H*,2*H*-perfluorooctyltrichlorosilane vapor (Alfa Aesar), overnight under vacuum.

To create microfluidic chips, polydimethylsiloxane (PDMS, SYLGARD 184, Dow Corning) mixed with curing agent at a ratio of 1 : 10 and degassed for 30 min. The PDMS was then poured on the abovementioned wafer and heated at 120 °C for 1 hour. Once cured, the polymer was peeled off the wafer and its inlets and outlets were punched using an 18-gauge blunt needle. Afterwards, the PDMS chip was bound to a glass slide using oxygen plasma. The inlets and outlets were fitted with 30-gauge poly(tetrafluoroethylene) (PTFE) tubing (Small Parts). All chip connections were sealed with PDMS.

### 2.6 Surface modification for affinity capture

Cell capture surfaces on straight herringbone channels (Fig. 1b and c) were modified using sandwich deposition method. 8 μL of biotinylated bovine serum albumin (biotin BSA) in T50 buffer (1 mg mL^−1^ in 10 mM Tris–HCl, pH 8.0, 50 mM NaCl) was injected into the channel and allowed to bind to channel for 45 min. Excess biotin BSA was pumped out with T50 followed by two pumps of air. Next, 8 μL neutravidin was injected to the channel and allowed to bind to biotin BSA for 15 min. Excess neutravidin was washed by injecting T50, DI water, and air, respectively. The coated chips were stored at 4 °C for not longer than a week before use. The affinity surfaces on the 5-channel herringbone chip (Fig. 1a) was coated using the abovementioned protocol except 15 μL of biotin BSA and 10 μL of neutravidin was used.

Prior to each experiment, straight herringbone channels were coated with 6 μL of anti-CD71 antibody. The 5-channel herringbone was coated using 10 μL of anti-CD71 to ensure maximal coating of all channels. Antibody coated chips were incubated for 20 min after which excess antibody was pumped out with air and stored in refrigerator until use.

### 2.7 Flow rate study

**Fig. 2:**
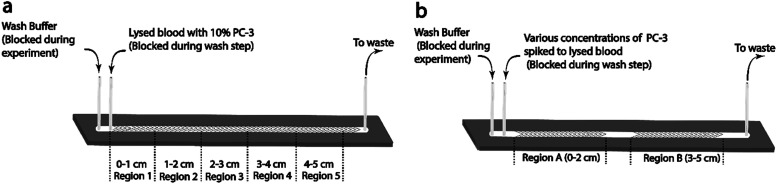
Schematic of the two single channel herringbone chips used for the flow rate study (a) and the PC-3 concentration study (b). Each chip was divided into regions based on distance from inlet.

**Fig. 3:**
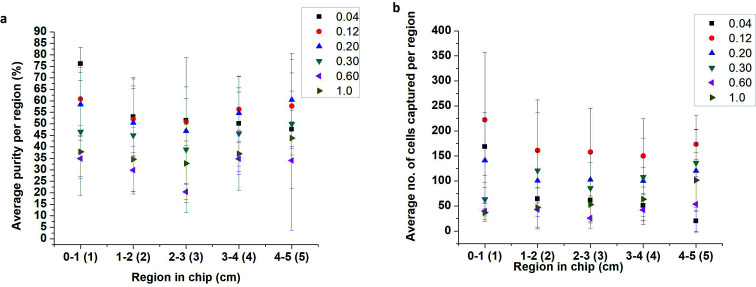
Graphs (a) show variation of capture purity with distance at different flow rates. Graphs (b) show the number of PC-3 cells captured per region at different flow rates. Flow rates range from 0.04–0.12 mL h^−1^. These graphs show that at lower flow rates, the number of cells captured is limited by the number of cells introduced to channel. However, the flow rates can only be increased until the shear acting on the cells do not exceed the antigen–antibody binding affinity. As a consequence of high shear, the cells captured reduce at higher flow rates. As a rule of thumb, the area closest to the inlet is most suited for cell enumeration for most flow rates, but at 0.12 mL h^−1^, the area closest to the outlet may also be used as an alternative (*n* = 3).

The effect of flow rate on cell capture was studied using lysed blood spiked with PC-3 cells at 10%. PC-3 spiked samples were flowed through the 5 cm single-channel herringbone chip at 0.04, 0.12, 0.20, 0.30, 0.60, and 1.00 mL h^−1^ flow rates for 30 min. The herringbone channel was divided into regions 1–5 based on its distance from the inlet (Fig. 2a). Each region was imaged, and cells were enumerated to determine the optimal flow rate to conduct subsequent studies and identify the regions of high purity (eqn (2)). The highest capture purity (76 ± 7% for region 1) of all the flow rates was observed for the 0.04 mL h^−1^ flow rate (Fig. 3a). However, the highest number of PC-3 cells were captured in all regions at 0.12 mL h^−1^ flow rate (Fig. 3b). Therefore, the PC-3 concentration studies were carried out using these two flow rates, as appropriate. Further, as the highest proportion of target cells were captured in the first 2 cm from the inlet (Fig. 3b) in the 5 cm herringbone chip, the PC-3 concentration studies were conducted using the 2 cm herringbone chip.

### 2.8 PC-3 concentration study

PC-3 cells were spiked into lysed blood at 20, 10, 1, 0.5, and 0.1%. Each sample was injected into the 2 cm single-channel herringbone chip (Fig. 1c) at 0.04 mL h^−1^ and allowed to flow for 30 min. The 2 cm herringbone chip had herringbone structures only in the 0–2 cm region (region A) and 3–5 cm (region B) region. Cell imaging and enumeration was performed for the Region A (Fig. 2b), as this region showed highest capture purities for this flow rate. Region B was not used for cell enumeration.

To observe the effect of higher cell input, a second set of 0.5 and 0.1% PC-3 spiked lysed blood samples were run through the same herringbone channel at 0.12 mL h^−1^ for 30 min. Region A was imaged and enumerated.

In order to evaluate the performance of the 5-channel herringbone chip (Fig. 1a), 0.1% PC-3 spiked lysed blood sample was flowed through the 5-channel herringbone chip. In this set of experiments, the wash buffer was made to flow in the reverse direction (outlet to inlet), to reduce background cell capture during the imaging step. The two channels showing the highest amount of cell capture were imaged.

### 2.9 MDA-MB-231 concentration study

MDA-MB-231 concentration study was conducted using the 5-channel herringbone chip (Fig. 1a). MDA-MB-231 cells were spiked into lysed blood samples at 10, 1, 0.5, and 0.1%. A flow rate of 0.20 mL h^−1^ was applied to the sample for 30 min. In theory, the flow rate in one channel is 0.04 mL h^−1^. However, the actual flow rate in a channel may vary as explained in Section 3.4. Each 1 cm channel has herringbone structures. Therefore, the two channels with the highest number of captured MDA-MB-231 cells were imaged and enumerated.

### 2.10 On-chip lysis experiment

300 μL of whole blood was incubated with PI for 20 min 100 μL of this whole blood sample was spiked with 10% of MDA-MB-231 cells. This sample was run through the L&B chip connected in series to the 5-channel herringbone chip (Fig. 1e). In this setup the flow rates for whole blood, DI water, and 3% BSA were 0.01, 0.02, and 0.09 mL h^−1^ respectively. Therefore, the theoretical flow rate in one of the herringbone channels is a little over 0.02 mL h^−1^. The remaining 200 μL of whole blood was lysed as described in Section 2.3 and spiked with 10% MDA-MB-231 cells. This sample was directly injected into a different 5-channel herringbone chip at 0.20 mL h^−1^. There are practical constraints to running whole blood and pre-lysed blood sample under comparable conditions, as described in Section 3.5. Therefore, lysed blood samples were only used to establish there was no significant difference between these blood samples and those described in Section 2.9.

### 2.11 Imaging

Affinity surfaces were imaged using a scientific CMOS camera (Quantalux, Thorlabs) coupled to an inverted epifluorescence microscope (Olympus IX71). Cell images and videos were analyzed using ImageJ software (Version 1.43u, National Institutes of Health). Cells entering the channel were recorded using a 4× objective lens with a 0.13 NA. A video recorded the number of cells entering the channel at the inlet for 3 min.

Unbound and weakly bound cells were washed with wash buffer before imaging capture regions. Cell images were taken using a 10× objective with 0.3 NA and the appropriate filter cube for each type of stained cell. Enrichment factor and capture purity was calculated using eqn (1) and (2).1
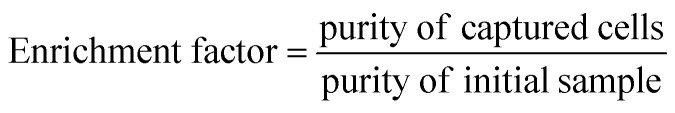
2
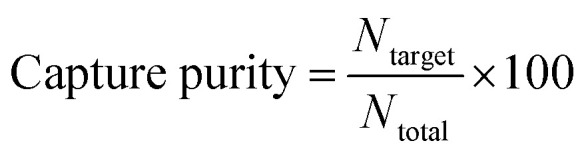
*N*_target_ indicates the number of target cells captured and *N*_total_ indicates the number of target cells and white blood cells captured.

## 3 Results and discussion

### 3.1 Flow rate study

For all flow rates, appreciable cell capture (Fig. 3b) occurs in regions 1 and 2 (Fig. 2a). However, at 0.04 mL h^−1^, in region 1, capture purity (eqn. (2)) is higher than all other rates. As the flow rate increases the region of the highest cell capture shifts towards the outlet. Slower flow rates allow better antigen–antibody contact resulting in more capture closer to the inlet while a higher number of effective collisions further down the channel enable cell capture at higher flow rates. Another important factor affecting cell capture, is the number of cells introduced to the channel. The highest cell capture for all regions is observed at 0.12 mL h^−1^ flow rate. However, increasing the flow rate also increases the number of non-target cells entering the channel, which consequently lowers purity near the inlet. In region 1 (Fig. 3a) purity at 0.04 mL h^−1^ (76 ± 7%) is higher than purity at 0.12 mL h^−1^ (61 ± 16%). But there is no significant difference between these values (*p* = 0.20 at *n* = 3 and *α* = 0.05). Since sample purity is an important consideration for subsequent analyses of an enriched sample, 0.04 mL h^−1^ rate was chosen to conduct PC-3 concentration studies.

### 3.2 PC-3 concentration study

**Fig. 4:**
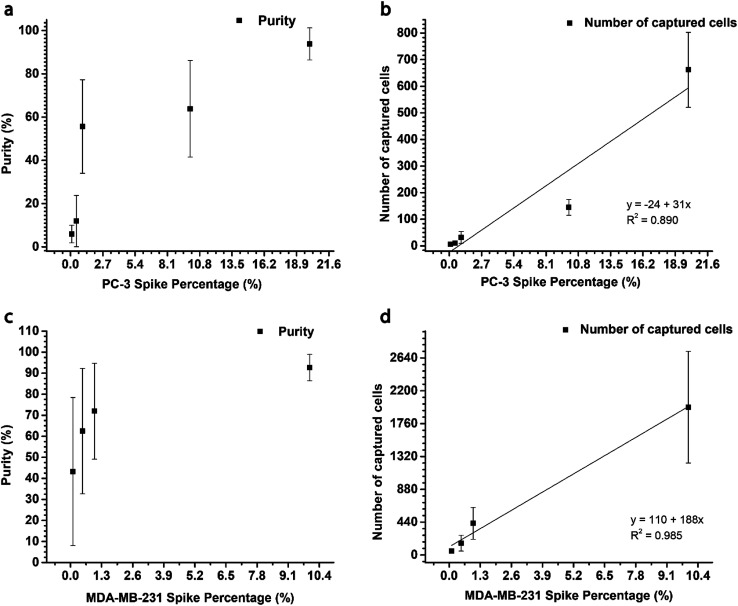
Results of the concentration studies done for PC-3 and MDA-MB-231 are shown in graphs a–d. (a) Graph of PC-3 spike percentage *vs.* capture purity (b) Graph showing the number of PC-3 cells captured at each spike percentage for concentration study using a 0.04 mL h^−1^ flow rate. Cancer cells exist as single cells and as clusters in the sample. Therefore, the number of captured PC-3 cells decrease when approaching 0.1% spike. In the PC-3 studies the introduction of a low number of target cells (0.04 mL h^−1^), limit the purity of enriched sample at lower spike percentages. (c) Graph of MDA-MB-231 spike percentage *vs.* capture purity. (d) Graph showing the number of cells captured at each spike percentage. Due to the high flow rate used in MDA-MB-231 studies (0.20 mL h^−1^) a large number of cells enter the channel every minute. Thus, we were able to capture MDA-MB-231 cells with a purity of 43 ± 35% even at a 0.1% spike of target cells. The sheer number of cells input at a 10% spike gives rise to non-specific binding close to the inlet. Hence, we captured an average of 2000 ± 700 cells within two channels, at 93 ± 6% purity. The total number of target cells captured far exceeds this value as there is significant cell capture in the other 3 channels as well (ESI 1 Video†) (*n* = 3).

The number of captured cells is seen to decrease as the spike percentage is lowered from 20% to 0.1% (Fig. 4b). However, the purity is maintained above 56% even at the 1% spike. Purity at 0.5% and 0.1% spikes are 12 ± 12% and 6 ± 4%, respectively, for region A (Fig. 2b). At a 0.1% spike PC-3 concentration in blood was between 1-3 cells per μL.

When 0.1% and 0.5% experiments were repeated at 0.12 mL h^−1^ flow rate the resulting purities were 2 ± 1% and 25 ± 6%, respectively. This data shows that at a spike percentage of 0.5, cell capture was limited by low cell input, but the 0.1% spiked sample is limited by chip design.

To determine the lowest detection limit of the 5-channel herringbone chip, the 0.1% spiked PC-3 sample was separated. To maintain a flow rate of 0.04 mL h^−1^ in the capture regions, the applied flow rate was set to 0.20 mL h^−1^. We were able to capture 11 ± 4 cells with a purity of 34 ± 5% using this method. At similar cell concentrations, PC-3 spiked samples showed lower target cell capture than MDA-MB-231 spiked samples. For 0.1% MDA-MB-231 spiked samples 55 ± 16 cells were captured, whereas analogous PC-3 spiked samples captured 11 ± 4 cells. The purity of PC-3 spiked samples was increased by flowing the wash buffer in the reverse direction. This will be discussed in detail in Section 3.4. Reversing the direction of the wash buffer should be done with caution as it will remove all weakly bound cells including target cells. In this study, the number of captured PC-3 cells was counted before reversing the flow. This allowed us to establish that captured target cells were not removed during the washing process. In Section 3.3 we observed that >1000 MDA-MB-231 cells were captured in the 10% spike. Non-specific binding of target cells also contributed to this value. In this instance reversing the flow may cause loss of target cells, but all specifically captured cells could be retrieved with much less background cells. Reversing the flow is a compromise between purity and captured target cell number (*N*_target_), therefore the decision to do so would depend on the subsequent analysis.

To determine the contribution of the antibody towards cell capture we ran a 0.1% PC-3 spiked lysed blood sample through a 5-channel herringbone chip without coating with anti-CD71. The chip captured 1 ± 2 cells with a purity of 17 ± 30% (*n* = 3). Therefore, we could conclude that in the absence of anti-CD71 cell capture would only occur due to aberrations in the channel structure. Further, the enrichment using the 2 cm single channel chip was 46 ± 32, whereas it was 339 ± 48 when using the 5-channel chip. By increasing the flow rate 5-fold we can achieve an enrichment that is higher than 5-fold (Table 1).

### 3.3 MDA-MB-231 concentration study

**Fig. 5:**
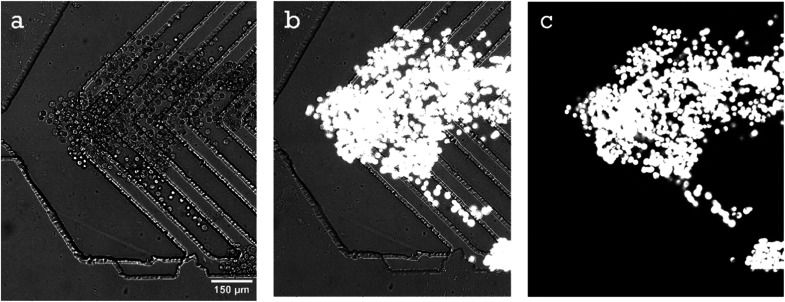
Image shows MDA-MB-231 (MDA) cells captured at the inlet of a 5-channel herringbone. Cells are captured from a 10% spiked sample where MDA cells are stained with MitoTracker Green. Both specific and non-specific binding of target cells is observed. The wash buffer is being flowed from left to right. (a) White light image of captured MDA cells. (b) White light and fluorescence image of MDA cells (white structures). (c) Fluorescence image of MDA cells.

**Fig. 6:**
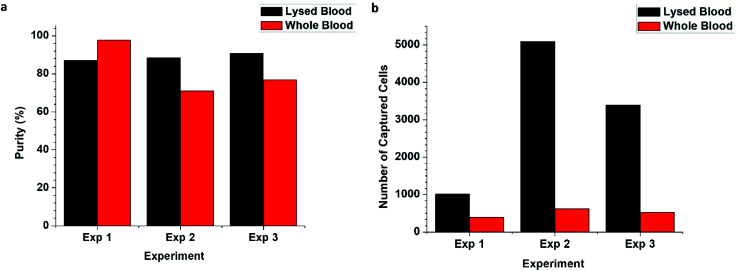
Graphs comparing the purity of on-chip lysed whole blood samples and lysed blood samples (a) and a plot of the number of cells captured in each sample (b). Each blood sample was spiked with 10% of MDA-MB-231 cells. The applied flow rate for lysed blood is 0.20 mL h^−1^ whereas the applied flow rate for whole blood was 0.01 mL h^−1^. Therefore, there is a large disparity in the number of cells captured. However, there is an average of 510 ± 120 cells captured in the whole blood setup, and its purity is not significantly different from that of the lysed blood setup (*p* = 0.447, *α* = 0.05, *n* = 3). There is also no significant difference (*p* = 0.400, *α* = 0.05, *n* = 3) between this set of pre-lysed blood samples and the ones used in the MDA-MB-231 concentration study.

At the 10% spike the 5-channel herringbone chip captured upwards of 1000 target cells (compare Fig. 4d and 6b). Therefore, we could capture target cells with a purity of 93 ± 6%. However, this level of capture is due to a combination of specific and non-specific binding of target cells. At high flow rates a large number of cell clusters enter the channel and the whole cluster is captured when at least one of those cells are specifically bonded to anti-CD71 (Fig. 5). This phenomenon is beneficial if the next step of the analysis requires captured cells to be subcultured. The capture purity progressively decreases as the spike percentage is lowered. This is a result of a constant number of background white blood cells and a low number of target cells. At a 0.1% spike MDA-MB-231 concentration in blood was reduced to 2 cells per μL. ESI1† (10% of MDA-MB-231 cells) and ESI2† (0.1% of MDA-MB-231 cells) show cell capture in the 5-channel herringbone chip.

Compared to PC-3 cells, MDA-MB-231 cells seem to have a higher rate of capture. At a 0.1% spike number of PC-3 cells captured was 11 ± 4 cells, whereas at the same spike percentage MDA-MB-231 showed a capture of 55 ± 16 cells. This difference can be due to inherent properties of the cells such as metastatic propensity and cell size. Pamler *et al.* has shown that cells with highly metastatic cells exhibit lower adhesion properties.^38^

### 3.4 5-Channel herringbone chip

Lower flow rates allow efficient target cell capture, but they also limited the number of input cells. Therefore, instead of using a single long 5 cm channel, 5 parallel 1 cm channels were created, so that the applied flow rate can be increased 5-fold while maintaining a lower flow rate at the affinity surfaces. The dimensions of the herringbone structure were based on other studies.^39,40^ The flow rate study showed that, at lower flow rates (below 0.12 mL h^−1^), most cell capture occurs near the inlet (Fig. 3b). Therefore, creating 5 parallel channels effectively increases the affinity surface area close to the inlet. Another advantage of using this channel is the ability to continue the experiment even when one channel is blocked. We can also choose the channel with the highest purity for subsequent analyses. There is also an increased probability of capturing rare cells in at least one of the channels. However, in this chip it is impossible to maintain a constant flow rate in all the channels. Excessive target cell capture (Fig. 5), debris, bubbles, and blocked channels effectively cause changes in pressure within the channels, thereby constantly affecting flow rates in all channels. At an applied flow rate of 0.20 mL h^−1^, the flow rate in a single channel ranged from 0.03–0.06 mL h^−1^.

Another interesting observation in this chip is that the distribution of cells in motion depended on the direction of the flow. When the flow direction was from the inlet to outlet cells were generally focused at the center of the channel (ESI3†). When the direction of flow was reversed the cells were generally focused closer to the walls (ESI4†). This is a result of the different pressure regimes operating within the channel brought about by the herringbone structures.^41^

### 3.5 On-chip lysis study

The on-chip lysis study was conducted to evaluate the compatibility of the 5-channel herringbone chip with the lysis circuit. A 10% spike of MDA-MB-231 cells was selected as this allowed reasonable number of target cells to flow to the affinity surfaces at 0.01 mL h^−1^. The flow rate at the affinity surfaces was a little over 0.02 mL h^−1^. The capture purity of the whole blood setup is 82 ± 14% whereas that of pre-lysed blood is 89 ± 2%. There is no significant difference in purity of the whole blood and lysed blood samples (*p* = 0.447, *α* = 0.05, *n* = 3). Further, the whole blood setup captured 510 ± 120 cells and achieved this purity using <7 μL of blood as opposed to the 100 μL of sample used in the lysed blood setup.

Given the non-Newtonian nature of whole blood, manipulating the flow rates of the whole blood setup to match the flow rates in the lysed blood setup is beyond the scope of this paper. Increasing the flow rate of blood to 0.20 mL h^−1^ would require a new chip design that allowed more time for complete erythrocyte lysis. The flow rate of BSA must be 9-fold higher than that of blood, therefore the flow rate at the affinity surfaces would be well above the threshold established in Section 3.1. Conversely, operating the lysed blood setup at 0.01 mL h^−1^ is not viable as the flow rate would be too low to transport sufficient number of cells to affinity surfaces. Also, the narrow 100 μm channel leading to the 1000 μm capture channels from inlet (Fig. 1a) introduces a backpressure to the lysis circuit which further complicates manipulating flow rates. Therefore, lysed blood samples were run at a flow rate of 0.20 mL h^−1^ and compared to the MDA-MB-231 concentration study. We established that there was no significant difference (*p* = 0.400, *α* = 0.05, *n* = 3) between these pre-lysed blood samples and those from the concentration study (Section 2.9).

## 4 Conclusion

In this study, we have successfully isolated prostate and breast cancer cells from blood, from concentrations as low as 1–3 cells per μL using CD71 as a capture target. CD71 shows potential use in tumor cell identification as well as target drug therapies. Further, we observed a 100-fold reduction in the limit of detection of a bare herringbone chip, and a 10-fold reduction in that of a nanoparticle coated herringbone chip described in our previous work. Therefore, we could infer that previous cell capture was limited by low sample input. Parallel flow chip designs can be employed to handle large sample inputs which can rapidly enrich target cancer cells from a patient sample. Table 1 shows that using our method cell enrichment becomes greater as the spike percentage decreases. These cells can then be used as seeds for cell cultures, which could subsequently be used for personalized drug testing among other clinical analyses. Coating the 5-channel herringbone chip with nanoparticles may lower the limit of detection further. The 5-channel herringbone chip can currently analyze 100 μL of blood and give a useful readout for the target cell concentrations as low as 1 cell per μL. However, this volume could be insufficient to detect target cells at lower concentrations.

The compatibility of the 5-channel chip with the whole blood lysis circuit opens a wide range of possibilities potentially allowing routine low-risk cancer screening at a very low cost and minimal preprocessing. Therefore, further experiments should be conducted to determine the flow dynamics of the lysis chip at different flow rate combinations. A modified design will allow the lysis circuit to handle larger volumes of blood without compromising purity. The combination of the lysis circuit with multi-channel capture chips could potentially be used to detect other non-cancerous cell types in blood.

## Conflicts of interest

The authors declare no conflict of interest.

## Supplementary Material

RA-010-D0RA03626A-s001

RA-010-D0RA03626A-s002

RA-010-D0RA03626A-s003

RA-010-D0RA03626A-s004

RA-010-D0RA03626A-s005
