# Supplementary material for: Tandem microfluidic chip isolation of prostate and breast cancer cells from simulated liquid biopsies using CD71 as an affinity ligand
Source: RSC Adv. 2020 Sep 2;10(54):32628–37. doi: 10.1039/d0ra03626a (PMC9056606; doi:10.1039/d0ra03626a)
Supplement: RA-010-D0RA03626A-s005 [file RA-010-D0RA03626A-s005.pdf]

Electronic Supplementary Material (ESI) for RSC Advances.
This journal is © The Royal Society of Chemistry 2020

Supporting Information

SI1 - Capture from 10% spike of MDA-MB-231 cells to lysed blood run at a rate of 0.20 mL/hr. The video shows the MDA cells fluorescing in white while being washed with 3% bovine serum albumin in phosphate buffered saline. The videos were recorded using a 4× objective lens with a 0.13 NA. The cells have been stained with MitoTracker Green.

SI2 - Capture from 0.1% spike of MDA-MB-231 cells to lysed blood run at a rate of 0.20 mL/hr. The video shows the MDA cells fluorescing in white while being washed with 3% bovine serum albumin in phosphate buffered saline. The videos were recorded using a 4× objective lens with a 0.13 NA. The cells have been stained with MitoTracker Green.

SI3 – The video shows the motion of white blood cells (fluorescing white) stained with propidium iodide. Flow direction from inlet to outlet at a rate of 0.20 mL/hr. The videos were recorded using a 4× objective lens with a 0.13 NA.

SI4 - The video shows the motion of white blood cells (fluorescing white) stained with propidium iodide. Flow direction from outlet to inlet at a rate of 0.20 mL/hr. The videos were recorded using a 4× objective lens with a 0.13 NA.
